# Metabolic Analysis of *Schizochytrium* Mutants With High DHA Content Achieved With ARTP Mutagenesis Combined With Iodoacetic Acid and Dehydroepiandrosterone Screening

**DOI:** 10.3389/fbioe.2021.738052

**Published:** 2021-11-18

**Authors:** Lei Zeng, Yanqi Bi, Pengfei Guo, Yali Bi, Tiantian Wang, Liang Dong, Fangzhong Wang, Lei Chen, Weiwen Zhang

**Affiliations:** ^1^ Laboratory of Synthetic Microbiology, School of Chemical Engineering & Technology, Tianjin University, Tianjin, China; ^2^ Frontier Science Center for Synthetic Biology and Key Laboratory of Systems Bioengineering (MOE), School of Chemical Engineering and Technology, Tianjin University, Tianjin, China; ^3^ SynBio Research Platform, Collaborative Innovation Center of Chemical Science and Engineering, Tianjin, China; ^4^ Center for Biosafety Research and Strategy, Tianjin University, Tianjin, China

**Keywords:** schizochytrium, DHA, metabolomic analysis, ARTP mutagenesis, iodoacetic Acid, dehydroepiandrosterone

## Abstract

High DHA production cost caused by low DHA titer and productivity of the current *Schizochytrium* strains is a bottleneck for its application in competition with traditional fish-oil based approach. In this study, atmospheric and room-temperature plasma with iodoacetic acid and dehydroepiandrosterone screening led to three mutants, 6–8, 6–16 and 6–23 all with increased growth and DHA accumulations. A LC/MS metabolomic analysis revealed the increased metabolism in PPP and EMP as well as the decreased TCA cycle might be relevant to the increased growth and DHA biosynthesis in the mutants. Finally, the mutant 6–23, which achieved the highest growth and DHA accumulation among all mutants, was evaluated in a 5 L fermentor. The results showed that the DHA concentration and productivity in mutant 6–23 were 41.4 g/L and 430.7 mg/L/h in fermentation for 96 h, respectively, which is the highest reported so far in literature. The study provides a novel strain improvement strategy for DHA-producing *Schizochytrium*.

## Introduction

Docosahexaenoic acid (DHA; C22:6, n-3) has been demonstrated to be beneficial to human health. It was reported that DHA is an essential component of human cerebral cortex and retina, and has roles in the nervous development of infants and children. It also has positive effect in the preventing cardiovascular diseases and anti-inflammatory and anti-tumor effects (Dessi et al., 2013). Marine fish is the traditional source of DHA, however, due to decrease in the number of marine fish and accumulate heavy metals and organic pollutants, many work focus on finding more sustainable and high-quality alternative approach of DHA source (Ganuza et al., 2008). Microalgae is well-known for DHA rich. Compared with other microalgae, thraustochytrid, a heterotrophic algae-like protist, *Schizochytrium* grows faster, accumulates more biomass and has higher DHA ratio in total fatty acid, furthermore, it also contains docosapentaenoic acid (DPA; C22:5, n-6), which is essential fatty acid ofhuman breast milk, the cortex of the human brain, and the retina of the eye (Makrides et al., 1994). Therefore, *Schizochytrium* has been used for industrial-scale production of DHA worldwide.

Since relatively low titer and productivity of DHA during fermentation process, high cost is a bottleneck for industrial DHA production using *Schizochytrium*. In recent years, several approaches, such as strain improvement, fermentation process optimization and genetic engineering, have been evaluated for increasing DHA titer and productivity in *Schizochytrium*. For example, 1) alleviate oxidative damage of *Schizochytrium* sp. HX-308 under high salinity by adaptive evolution resulted in improving lipid productivity by 1.96-fold (Sun et al., 2018); 2) By optimizing the oxygen supply strategy in the fermentation process, the DHA content and DHA yield of *Schizochytrium* M209059 were increased by 15.1 and 11.2%, respectively. (Ren et al., 2010); 3) overexpression of malonly-CoA:ACP transacylase led to 81.5% increase of DHA titer after a glucose fed-batch fermentation in *Schizochytrium* sp. MYA1381 (Li et al., 2018). Up to now, the highest report in the literature showed that *Schizochytrium* sp. 31 produced 151.4 g/L of dry cell weight and 28.9 g/L of DHA after 96 h fermentation, when using glycerol as carbon source (Chang et al., 2013). Nevertheless, more efforts are still needed for improving DHA titer and productivity for large-scale industry application.

Compared with other breeding methods, atmospheric and room temperature plasma (ARTP) technology, which is based on radio-frequency atmospheric-pressure glow discharge plasma, has many advantages, such as high positive efficiency and more table mutants (Wang et al., 2014; Ottenheim et al., 2018). Therefore, it has been widely applied to marine microorganisms breeding including *Schizochytrium sp*., resulting in identifying mutants with increasing DHA and biomass accumulation in recent years (Yuan et al., 2015; Zhao et al., 2018; Li et al., 2020). For example, a novel approach of ARTP mutagenesis coupled with malonic acid and zeocin screening was developed to obtain *Schizochytrium* mz-17. After Fe^2+^ supplementation in fed-batch fermentation, DHA titer reached 14.0 g/L, which was two-fold higher than the wild type (Zhao et al., 2018).

It was proposed that *Schizochytrium* might likely utilize polyketide synthase systems for DHA production (Metz et al., 2001). The acetyl-CoA producing pathway, NADPH producing pathway, fatty acid synthase (FAS) are important metabolic modules for achieving efficient DHA accumulation in marine microorganisms (Qiu et al., 2020). Several screening agents targeting these metabolic modules have been evaluated in *Schizochytrium* (Supplementary Table S1), including the iodoacetic acid inhibiting Embden-Meyerhof-Parnas (EMP) pathway (Chance and Park, 1967), and malonic acid weakening tricarboxylic acid (TCA) cycle (Beevers, 1952), leading to decreased acetyl-CoA formation and utilization, respectively. An iodoacetic acid-resistant and malonic acid-resistant mutant named *Schizochytrium sp*. HX-308M, which was proved enhancing acetyl-CoA supply, was obtained. The lipid concentration and DHA ratio in total fatty acid of *Schizochytrium* sp. HX-308M were increased 34.8 and 38.9% (Lian et al., 2010). Two *Schizochytrium* mutants named OUC002 and OUC007 were also achieved based on their resistance to quizalofop-p-ethyl, a FAS inhibitor. The DHA concentrations of *Schizochytrium* OUC002 and OUC007 were elevated by 13.7 and 28.8%, comparing with the wild type (Xu et al., 2012). The 2′, 2′-bipyridine can induce ROS pathway, resulting in death of strains with weaker antioxidant capacity. *Schizochytrium* mutant survived in plates containing 2′, 2′-bipyridine exhibited 29.8% improvement of DHA titer, in comparison with the wild type (Yuan et al., 2015). In addition, overexpression of malic enzyme coding gene to strengthen NADPH supply could also elevate DHA titer in *Schizochytrium* (Wang et al., 2019). However, so far, few reports have focused on agents targeting NADPH-producing pathway for microalgal breeding. Dehydroepiandrosterone is an adrenal hormone used to inhibit the activity of glucose-6-phosphate dehydrogenase, a key enzyme that catalyzes NADPH supply, in bovine serum red blood cells (Tian et al., 1999). The roles of glucose-6-phosphate dehydrogenase in improving DHA and lipid accumulation were also previously demonstrated in marine microorganisms (Cui et al., 2016; Xue et al., 2017; Xue et al., 2018). For example, enhanced glucose-6-phosphate dehydrogenase expression could lead to more DHA production in *Aurantiochytrium sp*. SD116, closely related specie of *Schizochytrium*, but the growth was significantly decreased (Cui et al., 2016). However, there is no report whether dehydroepiandrosterone can be used for screening agents in marine microorganisms.

In this study, an integrated ARTP mutagenesis and dehydroepiandrosterone and iodoacetic acid-based screening method was applied to *Schizochytrium* ATCC 20888, resulting in several mutants with increased growth and DHA content. In addition, a LC-MS based metabolomics was carried out to study mechanisms associated with improved growth and DHA content in the mutants. The new findings could be valuable for the better understanding of *Schizochytrium* metabolism and provide valuable information regarding regulatory targets for engineering *Schizochytrium* for even high DHA titer in the future.

## Materials and Methods

### Strains and Chemicals

*Schizochytrium* ATCC 20888 was purchased from the American Type Culture Collection (ATCC). Sea salt, fatty acid standard and antifoam SE-15 were purchased from Sigma-Aldrich (St. Louis, MO, United States). Yeast extract was purchased from OXOID (Basingstoke, United Kingdom). All the other chemicals were purchased from Jiang Tian Chemical Technology Co., Ltd., (Tianjin, China).

### Cultivation

For shaking-flask cultures, strain grown at basal liquid medium for 2 days was used as seed cultures. Approximately 0.95 OD_660_ or 0.004 g dry cell weight (DCW) of seed cultures were transferred into 20 ml of rich medium and cultivated at 28°C and 180 rpm for 3 days. OD_660_ was determined by a UV-1750 spectrophotometer (Shimadzu, Japan).

Fed-batch culture was carried out in a 5-L bioreactor with an initial volume of 2.2 L fermentation medium equipped with automatic controls of agitation, temperature, airflow, pH and dissolved oxygen concentration. The seed culture grown in rich medium was transferred into a 5 L fermentor at an inoculum size of 10% (*v*/*v*) (Approximately 950 OD_660_ or 4 g DCW). The temperature controlled at 28°C. The dissolved oxygen levels were kept above 30%. The pH was maintained at 6.5 ± 0.1 by automated addition of 0.5 M H_2_SO_4_. A 60% (*w*/*v*) glucose solution was fed into the fermentation medium to maintain glucose concentration at 20–50 g/L (Qu et al., 2013a).

The composition of basal, rich and fermentation medium followed the previous study (Zhao et al., 2017; wang et al., 2019). The composition of 1 L basal liquid includes 5 g glucose, 1 g peptone, 1 g yeast extract and 20 g sea salt. Rich medium containing 40 g/L glucose, 10 g/L yeast extract, 1 g/L (NH_4_)_2_SO_4_, 4 g/L K_2_HPO_4_·2H_2_O, 12 g/L Na_2_SO_4_, 10 g/L Mg SO_4_·7H_2_O, 7 g/L K_2_SO_4_ and 2 g/L KCl. The fermentation medium is the same as the rich medium except that the concentration of glucose and yeast extract is100 g/L and 25 g/L.

### Assay of *Schizochytrium* sp. 31 Sensitivity to Selected Agents

One hundred microliters of exponential phased *Schizochytrium sp*. 31 were spread on solid plates containing 0, 20, 60, 100, 160, 180, 200 mg/L of iodoacetic acid (IAA) or 0, 10, 20, 30, 40, 50, 60, 70 mg/L of dehydroepiandrosterone (DHEA) or 0, 10, 20, 30, 40, 50, 60, 70, 80 mg/L of IAA and DHEA (IAA and DHEA at a ratio of 4:1), respectively, and then cultivated at 28°C until the colony was no longer become larger (3 days) (Zhao et al., 2018). A minimal three replicated plates were conducted for determination of the selective concentration.

### Atmospheric and Room Temperature Plasma Mutagenesis of *Schizochytrium*

ARTP mutagenesis of *Schizochytrium* ATCC 20888 was carried out according to the methods with minor modification on the ARTP treated time (Liu et al., 2017). Briefly, approximate 10^6^–10^7^ of exponential phased cells were inoculated to a sterilized sample plate, exposed to an ARTP breeding mutagenesis machine (a Model ARTP-M, Yuan Qing Tian Mu Biotechnol Inc., Wuxi, China) for 0, 20, 40, 60, 80, 100, or 120 s, and then quickly transferred to basal medium. Fifty microliters of diluted cultures were inoculated on basal medium plates and incubated until the colony occurred. The optimized ARTP treated time was measured by the lethal rate. The mutants were screened on basal medium plates supplemented with 40 g/L iodoacetic acid and 10 g/L dehydroepiandrosterone. The lethality rate was determined as follows:

$$\text{Lethality rate }\left(\text{\%}\right)=\frac{\text{control colonies}-\text{suvival colonies}}{\text{control colonies}}\times 100\text{\%}$$ (1)

The individual colonies with ARTP treatment of 0 s for control colonies and each other ARTP treated colonies were counted, respectively. (Zhao et al., 2018).

### Biomass, Glucose, Fatty Acids and Specific Growth Rate Determination

Biomass determination of supernatant followed the previous study (Li et al., 2017). 2 ml of fermentation broth was centrifuged at 8,000 × *g* for 5 min, washed once with distilled water, and vacuum freeze-dried for 12 h to determine the DCW. Glucose in the supernatant was determined with glucose oxidase method (De Swaaf et al., 1999) using Glucose Oxidase Assay Kit (Biosino Biotechnology and science Co., Ltd., China). Fatty acid contents were measured according the methods described (Wang et al., 2019). Briefly, 20 mg of lyophilized cell powder was added with 2 ml of chloroform, 2 ml of methanol containing 3% (*v*/*v*) sulfuric acid and 0.5 mg/L nonadecanoic acid (an internal standard). The reaction was performed at 97°C for 2 h, and added 1 ml of distilled water to separate the water phase from chloroform phase. The extracted chloroform phase was used for fatty acid determination by a GC–MS system-GC 7890 coupled to an MSD 5975 (Agilent Technologies, Inc., Santa Clara, CA) equipped with a HP-5MS capillary column (30 m × 250 mm id). The fatty acids content was calculated using standard curve methods with nonadecanoic acid as an internal standard. The specific growth rate was calculated by the equation, *μ* (h^−1^)=(ln*X*_2_-ln*X*_1_)/(*t*_2_-*t*_1_), where *X*_1_ and *X*_2_ are the cell dry weight (g/L) at the time *t*_1_ and *t*_2_, respectively (Sun et al., 2016).

### Determination of Glucose-6-Phosphate Dehydrogenase and 3-Phosphoglyceraldehyde Dehydrogenase Enzyme Activities

The preparation of cell homogenates was as follows. Briefly, 1 ml of cells were harvested at 12, 24, 36 or 72 h, added with 1 ml of extract solution, and then disrupted by cell disruptor. The solution was centrifuged 8,000 *g* for 10 min at 4°C. The supernatant was extracted for protein concentration and enzymatic activities analysis. The protein concentration was measured by Bradford method (Nouroozi et al., 2015) using Bradford Protein Assay Kit (Sangon Biotech. Co., Ltd., Shanghai, China). The glucose-6-phosphate dehydrogenase and 3-phosphoglyceraldehyde dehydrogenase activity followed the previous study (Langdon, 1966; Zwickl et al., 1990). Briefly, the glucose-6-phosphate dehydrogenase or 3-phosphoglyceraldehyde dehydrogenase activity was determined spectrophotometrically in 340 nm by monitoring the rate of NADPH or NADH formation at 30°C. The determination of the activity of glucose-6-phosphate dehydrogenase and 3-phosphoglyceraldehyde dehydrogenase followed the instructions of 6-Phosphate Dehydrogenase Activity Assay Kit (Nanjing Biobox Biotech. Co., Ltd., Nanjing, China) and Glyceraldehyde 3-Phosphate Dehydrogenase Activity Assay Kit (Nanjing Biobox Biotech. Co. Ltd., Nanjing, China). The activity of glucose-6-phosphate dehydrogenase or 3-phosphoglyceraldehyde dehydrogenase was normalized by the protein concentration.

### Comparative LC-MS Metabolomics Analysis

Samples preparation for LC-MS metabolomics analysis followed the previous study (Li et al., 2015). Briefly, cells grown at the 24 or 48 h were centrifuged at 8,000 × *g* for 5 min at 25°C (Eppendorf 5430R, Hamburg, Germany). The supernatant was discard. The sediment was resolved in 900 µL of solution 1 (80:20 MeOH/H_2_O, stored at −80°C), quickly frozen in liquid nitrogen, and thawed on dry ice to release metabolites (Park et al., 2011). The cells were frozen-thrawed for three times to release the whole metabolites, and the supernatants were collected by centrifugation at 150,00 × *g* for 5 min at 4°C (Park et al., 2011). The left cell debris were then re-suspended in solution 1 and the above extraction process was repeated. The twice supernatants were mixed and stored at −80°C until LC-MS analysis. An Agilent 1,260 series binary HPLC system (Agilent Technologies, Waldbronn, Germany) using a SYnergi Hydro-RP (C18) 150 mm × 2.0 mm I.D., 4 μm 80 Å 549 particles column (Phenomenex, Torrance, CA, United States), coupled to an Agilent 6410 550 triple quadrupole mass analyser equipped with an electrospray ionization source was used for LC-MS analysis. Data processing and statistical analysis were conducted according to the method described previously (Wang et al., 2019).

### Statistical Analysis

Two-tailed Student’s *t*-tests were carried out for significant difference. A minimal three replicates were carried out, and *p* < 0.05 was considered significant different.

## Results

### Atmospheric and Room Temperature Plasma Mutagenesis With Iodoacetic Acid- and Dehydroepiandrosterone-Based Screening

**FIGURE 1:**
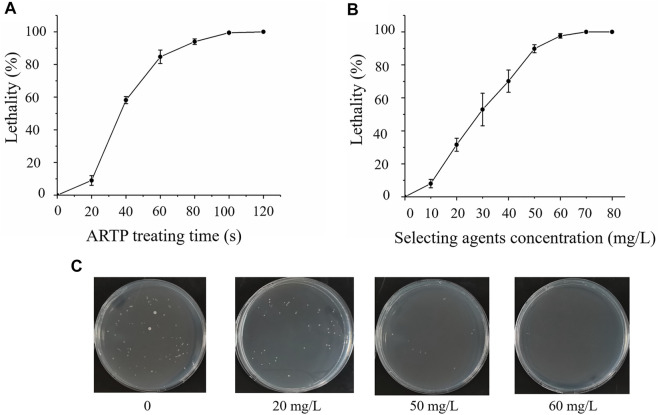
Screening and sensitivity of *Schizochytrium* ATCC20888 to selected agents. **(A)** Atmospheric and room temperature plasma (ARTP) treatment time; **(B)** Sensitivity of *Schizochytrium* ATCC 20888 to selected agents (iodoacetic acid (IAA) and dehydroepiandrosterone (DHEA) at a ratio of 4:1); **(C)** Sensitivity of *Schizochytrium* ATCC 20888 on solid plates supplemented with iodoacetic acid and DHEA at a ratio of 4:1. Error bar represents standard deviation of three biological replicates.

*Schizochytrium* ATCC 20888 cells were mutated with ARTP for varying times with a lethal rate as the selected parameter. As shown in Figure 1A, the optimal treatment was 60 s with a lethal rate of 90%. It was previously reported that strengthening pentose phosphate pathway (PPP) results in improving total fatty acid content with sacrificing biomass accumulation in marine microorganisms (Cui et al., 2016; Xue et al., 2018), while enhancing EMP could improve growth of marine microorganisms (Lian et al., 2010; Sun et al., 2020). Therefore, iodoacetic acid or DHEA, an inhibitor of EMP or PPP pathways, was applied to determine the sensitivity of *Schizochytrium* ATCC 20888. The results showed that the lethal rate of *Schizochytrium* ATCC 20888 reached 90% when the concentration of iodoacetic acid and DHEA was over 160 and 40 mg/L, respectively (Supplementary Figures S1A, S1B). The iodoacetic acid was combined with dehydroepiandrosterone at the concentration ratio of 4:1 for screening on the plates. It was found that the growth of *Schizochytrium* ATCC 20888 was significantly inhibited over 90% when the concentration of both selected agents together was over 50 mg/L (iodoacetic acid: 40 mg/L; DHEA: 10 mg/L) (Figures 1B,C).

The mutagenesis was carried out using the above optimal parameters. Mutants were first screened based on colony size, and then confirmed by comparative DHA concentration and growth analysis after cultivating these mutants in flask cultures. The analysis showed that mutant 6 was the highest in terms of the dry cell weight or DHA concentration, which was 1.1- or 1.4-fold higher than that of the wild type ATCC 20888 (Supplementary Figure S2A; Supplementary Table S2). In addition, only mutant 6 could survive on plates supplemented with increased iodacetic acid and DHEA concentration. The whole process was then repeated with mutant 6 as the starting strain, and the concentration of iodacetic aicd and DHEA was further increased up to 16 mg/L and 64 mg/L, respectively. As shown in Supplementary Figure S2B, we identified that DCW of mutant 6–8, 6–16, or 6–23 from the 25 second-round mutants analysed was significantly higher than that of mutant 6 from the first round. The DCW was elevated by 1.1-, 1.1- or 1.2-fold in mutant 6–8, 6–16 or 6–23, respectively. The DHA concentration of mutant 6–8, 6–16, or 6–23 was improved by 1.5-, 1.4- or 1.6-fold, respectively (Supplementary Table S3). In addition, the DPA concentration of mutant 6–23 was improved by 1.4-fold, while it was decreased by 38.4% or 40.4% in mutant 6–8 or 6–16. At last, mutant 6–8, 6–16 and 6–23 were sub-cultivated for 20 more times, the DCW and DHA titre of the 20^th^ passages were compatible to these of the first passages, suggesting the stability of mutant 6–8, 6–16 and 6–23 (Supplementary Figure S3).

### Characterization of Mutant 6–8, 6–16, and 6–23 in Shake-Flask Culture

**FIGURE 2:**
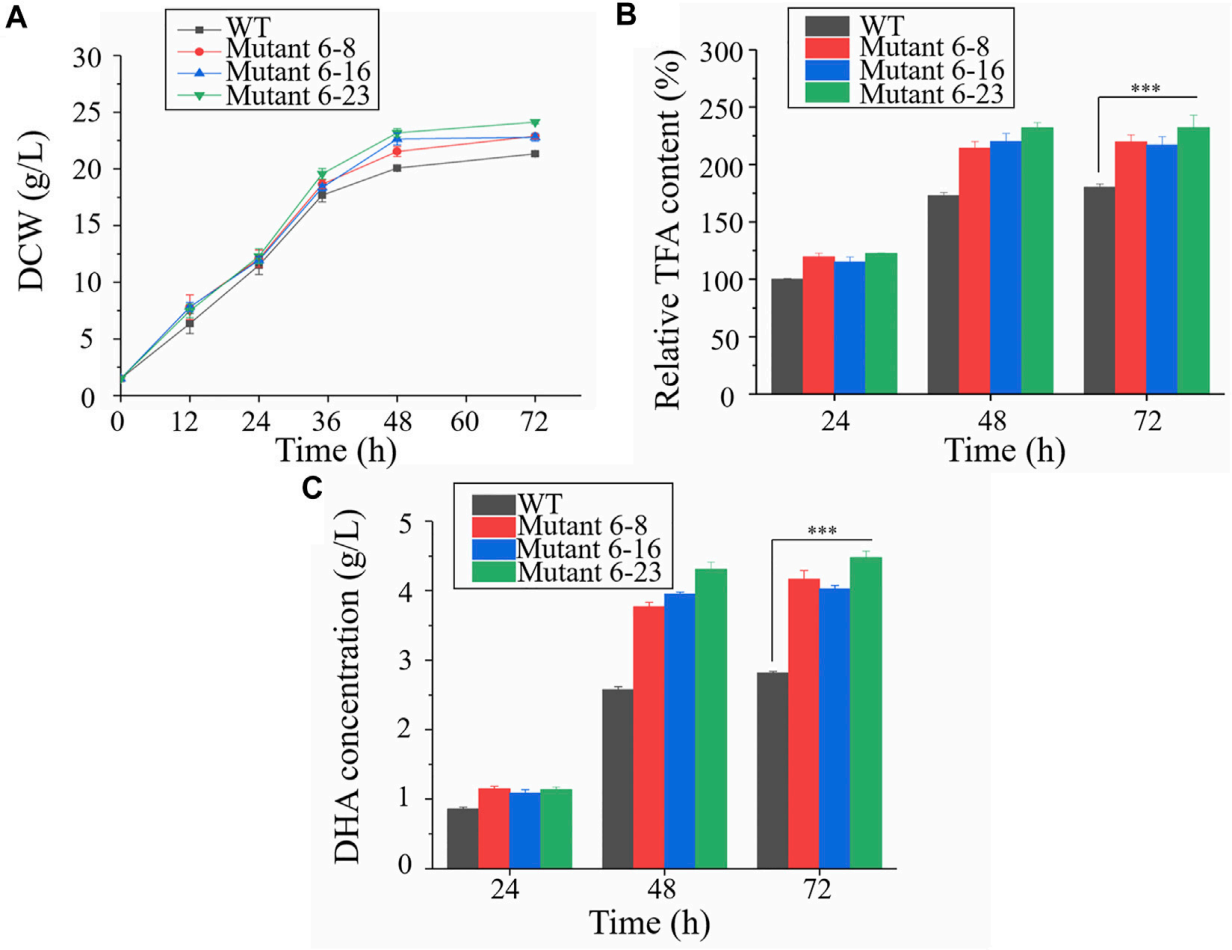
Comparison of biomass accumulation, total fatty acid and DHA concentration in shake-flask cultures. Black represents the wild type; red represents mutant 6–8; blue represents strain 6–16; green represents mutant 6–23, respectively. **(A)** Biomass accumulation; **(B)** Total fatty acid. The total lipid content in the wildtype at 24 h was set as 100%; **(C)** DHA concentration. WT, wild type; DCW, dry cell weight ; TFA, total fatty acid. Error bar represents standard deviation of three biological replicates. Asterisks indicate significant difference between the control and stress treatments based on Student’s *t*-tests (∗*p* < 0.05; ∗∗*p* < 0.01; ∗∗∗*p* < 0.005).

**FIGURE 3:**
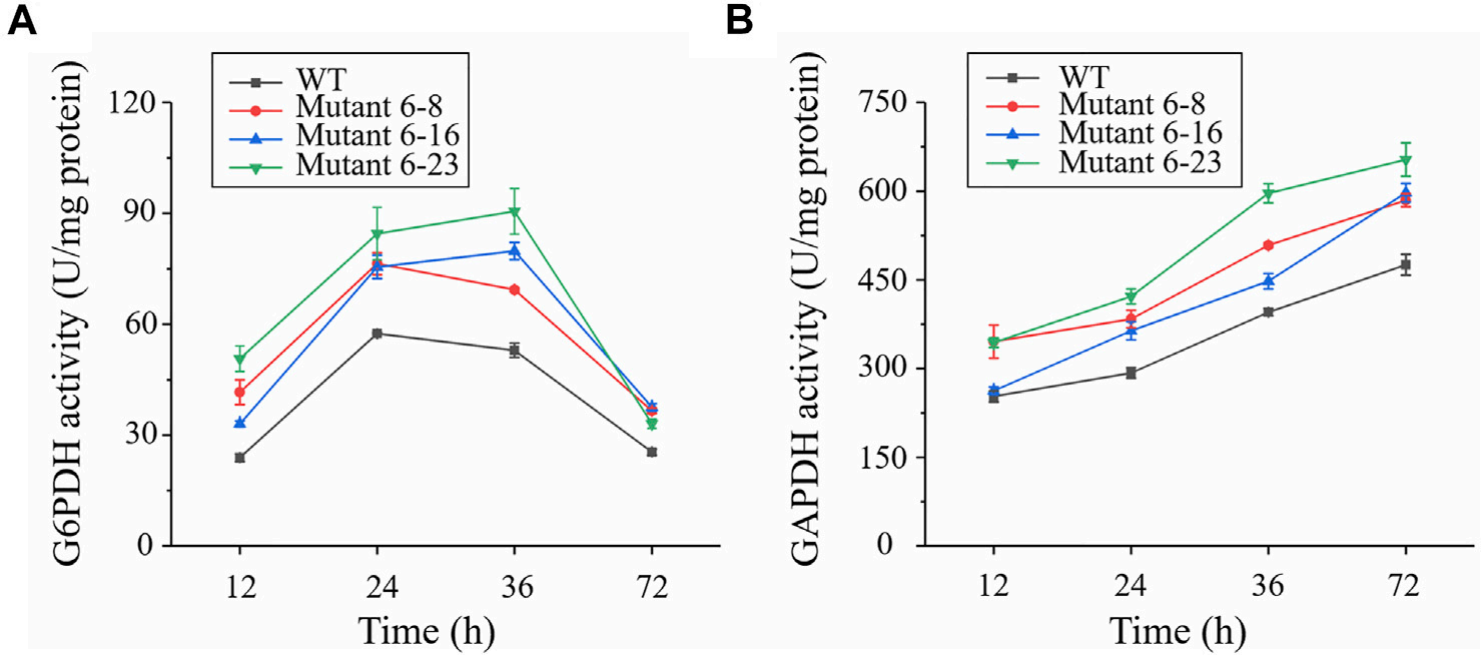
Comparison of glucose-6-phosphate dehydrogenase and phosphoglyceraldehyde dehydrogenase in shake-flask cultures. Black represents the wild type; red represents strain 6–8; blue represents mutant 6–16; green represents mutant 6–23, respectively. **(A)** Enzymatic activity of glucose-6-phosphate dehydrogenase (G6PDH); **(B)** Enzymatic activity of phosphoglyceraldehyde dehydrogenase (GAPDH). Error bar represents standard deviation of three biological replicates.

Approximately 0.95 OD_660_ or 0.004 g dry cell weight (DCW) of the wild type and mutant 6–8, 6–16, and 6–23 seed cultures were inoculated into shake flasks to compare the growth, enzyme activities and fatty acid concentration. It was shown in Figure 2, mutant 6–8, 6–16, and 6–23 accumulated much more biomass at the stationary growth phase, compared with the wild type. The specific growth rate of wild type, mutant 6–8, 6–16, and 6–23 were *μ* = 0.011, 0.012, 0.012, 0.013 h^−1^ during logistical growth phase, respectively. Mutant 6–23 performed better than other mutants, as its dry cell weight was 1.2-fold higher than the wild type at the 72 h. The activities of phosphoglyceraldehyde dehydrogenase and glucose-6-phosphate dehydrogenase, which were the targets of iodoacetic acid and DHEA, respectively, were determined. As shown in Figure 3A, the activity of glucose-6-phosphate dehydrogenase was the highest at the 24 h for wild type and mutant 6–8, and it was at the 36 h for mutant 6–16 or 6–23. The activity of glucose-6-phosphate dehydrogenase in all strains was decreased when it reached the peak. Comparing with glucose-6-phosphate dehydrogenase, the activities of phosphoglyceraldehyde dehydrogenase were increased with cultivation time course on in all strains (Figure 3B). The phosphoglyceraldehyde dehydrogenase and glucose-6-phosphate dehydrogenase activities in the mutants were both increased 1.3-fold over the wild type (Figures 3A,B) at all selected time points, which further confirmed the effectiveness of iodoacetic acid and DHEA-based screening. The most significant increase in phosphoglyceraldehyde dehydrogenase and glucose-6-phosphate dehydrogenase activities was found in mutant 6–23, which was 71.0 and 37.4% higher than the wild type at 72 h, respectively.

Cells were harvested at the exponential and stationary phases to compare total fatty acid content. The results showed that the total fatty acid contents of mutant 6–8, 6–16 and 6–23 were significantly enhanced than that of the wild type at the 48 and 72 h, respectively, among which mutant 6–23 accumulated the most amount of fatty acids, 1.2-, 1.2-, 1.3-fold higher than mutant 6–8, 6–16 and the wild type at the 72 h, respectively. The fatty acid contents of mutants and the wild type were also comparatively determined. Consistent with the results of phosphoglyceraldehyde dehydrogenase and glucose-6-phosphate dehydrogenase activities, DHA concentrations of mutant 6–8, 6–16 and 6–23 were also significantly enhanced compared to the wild type at both 48 and 72 h (Figure 2C). The DHA concentration in mutant 6–23 was the highest, reaching a level of 4.6 g/L at 72 h. Therefore, it was likely to suggest the correlation between DHA content and activities of dehydrogenase and glucose-6-phosphate dehydrogenase.

### Comparative Metabolomic Analysis Between Mutants and the Wild Type

Metabolomics, which was for identification and quantitation of metabolites in biological reaction, is a powerful technology to compare the metabolic status in different biological samples (Zhou et al., 2012). Among these technologies, the coupling of liquid chromatography to mass spectrometry (LC-MS) has gradually become an important tool to quantitative analysis of central carbon metabolites since its high throughput, good metabolites coverage and soft ionization (Zhou et al., 2012). The LC-MS has been recently applied to many marine microorganisms, including explaining chemodiversity of a rich marine microorganism tropical ecosystem (Reveillon et al., 2019), identifying hub metabolites relevant to resist Fe^2+^ and high light stress condition in *Haematococcus pluvialis* (Su et al., 2014), and exploring possible mechanisms of elevating fatty acid contents after overexpressing *EL O3* gene in *Schizochytrium sp*. 31 (Wang et al., 2019). In all, LC-MS can provide accurate metabolic changes in marine microorganisms.

In this study, cells of mutant 6–8, 6–16, 6–23 and WT were harvested at the exponential (24 h) and stationary phases (48 h) and then subjected to LC-MS based metabolomic analysis (Supplementary Table S4). The principal component analysis (PCA) was used to judge the quality of the LC-MS metabolomics (Supplementary Figure S4). It was shown that 1) a good reproducibility of each sample as three biological replicates are obviously clustered together; 2) an apparent metabolic change occurred among mutant 6–8, 6–16, 6–23 and the wild type since samples of these mutants were visibly separated in the PCA plots at both points; 3) the most significant metabolic changes occurred between wild type and mutant 6–8 or 6–16, because of samples of mutant 6–8 and 6–16 far away from the wild type at both 24 and 48 h.

**FIGURE 4:**
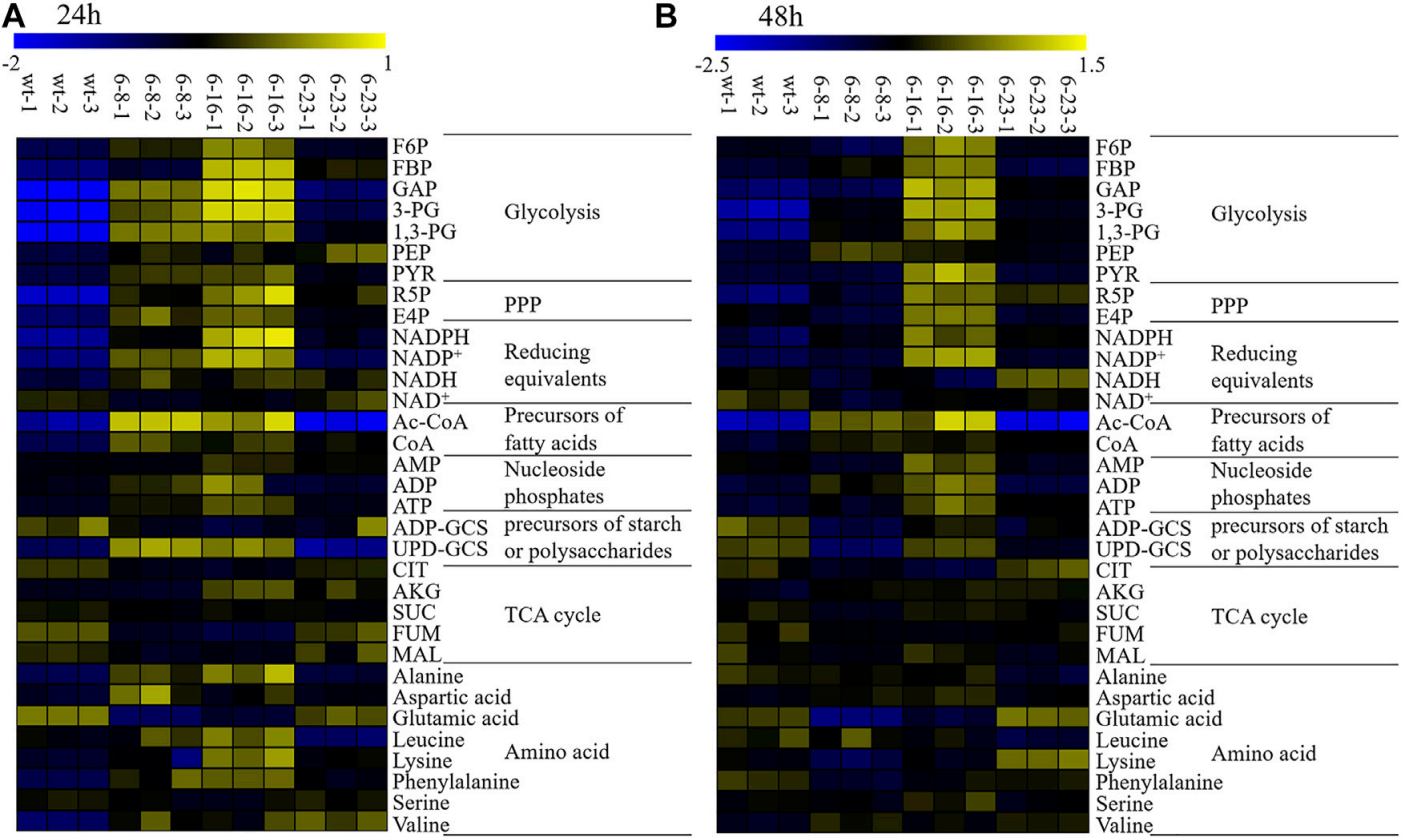
Heatmaps of LC–MS targeted metabolomics of the wild type and mutants. NADPH, nicotinamide adenine dinucleotide phosphate; NADP^+^, oxidized form of nicotinamide adenine dinucleotide phosphate; NAD^+^, nicotinamide adenine dinucleotide; NADH, Nicotinamide adenine dinucleotide; ADP-GCS, adenosine 5′- diphosphoglucose; UDP-GCS, uridine 5′-diphosphoglucose; ATP, adenosine triphosphate; ADP, adenosine diphosphate; CoA, Coenzyme A hydrate; AMP, adenosine monophosphate; FBP, fructose 1,6-bisphosphate; F6P, fructose 6-phosphate; R5P, ribose 5-phosphate; E4P, erythorse 4-phosphate; CIT, citricric acid; 3-PG, 3-phosphoglyceric acid; 1,3-PG, 1,3-phosphoglyceric acid; GAP, glyceraldehyde 3-phosphate; PEP, phosphoenolpyruvic acid; GLU, glutamic acid; PYR, pyruvic acid ;AKG, 2-oxoglutaric acid; MAL, malate acid; SUC, succinic acid; FUM, fumaric acid; Ac-CoA, Acetoacetyl coenzyme A. **(A)** WT, mutant 6–8, mutant 6–16 and mutant 6–23 at 24 h; (B) WT, mutant 6–8, mutant 6–16 and mutant 6–23 at 48 h. Three biological replicates were carried out.

Heat maps is a popular tool to display information-rich data in two or three dimensions, and a visualization route to represent the relative abundance of ions in samples which is described with color intensity (Ivanisevic et al., 2015). It allows for sample classification and the description of features that are driving the classification by adding dimension of data visualization (Babicki et al., 2016). It has been well used for exploring metabolic flux of microalgae previously (Wang et al., 2019; Lv et al., 2020). In this study, heatmaps of targeted metabolite analysis in all samples were generated for better interpreting the qualitative information of these metabolites. The results were shown in Figure 4.

The metabolomic analysis showed similar metabolic changes among these mutants, including: 1) EMP seemed strengthened as the up-regulation of 3-PG, 1,3-PG, PYR at both 24 and 48 h was observed. EMP is known to produce ATP, Ac-CoA, NADH and which are necessary substances for biomass accumulation (Chen et al., 2017); 2) PPP seemed enhanced, as it key metabolite R5P were up-regulated at both 24 and 48 h. One of alternative NADPH-producing sources is PPP. It was previously reported that elevation expression of PPP leads to significant increase of total fatty acid content in *Yarrowia lipolytica* or *Aurantiochytrium sp*. SD116 (Cui et al., 2016; Xu et al., 2017; Liu et al., 2019); 3) The lipogenesis pathway seemed up-regulated, as CoA and NADPH, the precursors of lipid biosynthesis, were up-regulated; 4) TCA cycles seemed attenuated, as metabolites involved in TCA cycles, such as CIT, MAL, SUC, FUM at 24 and 48 h in M-6-8; CIT, FUM at 24 and 48 h in M-6-16; CIT, SUC at 24 h or MAL at 48 h in M-23, were down-regulated. Pushing carbon source of TCA cycle into lipogenesis could significantly increase lipid accumulation in oleaginous yeast *Y. lipolytica* or *Saccharomyce cerevisiae* (Silverman et al., 2016; Yu et al., 2018).

### Evaluation of Application Potential of Mutant 6–23 in Fermentor

**FIGURE 5:**
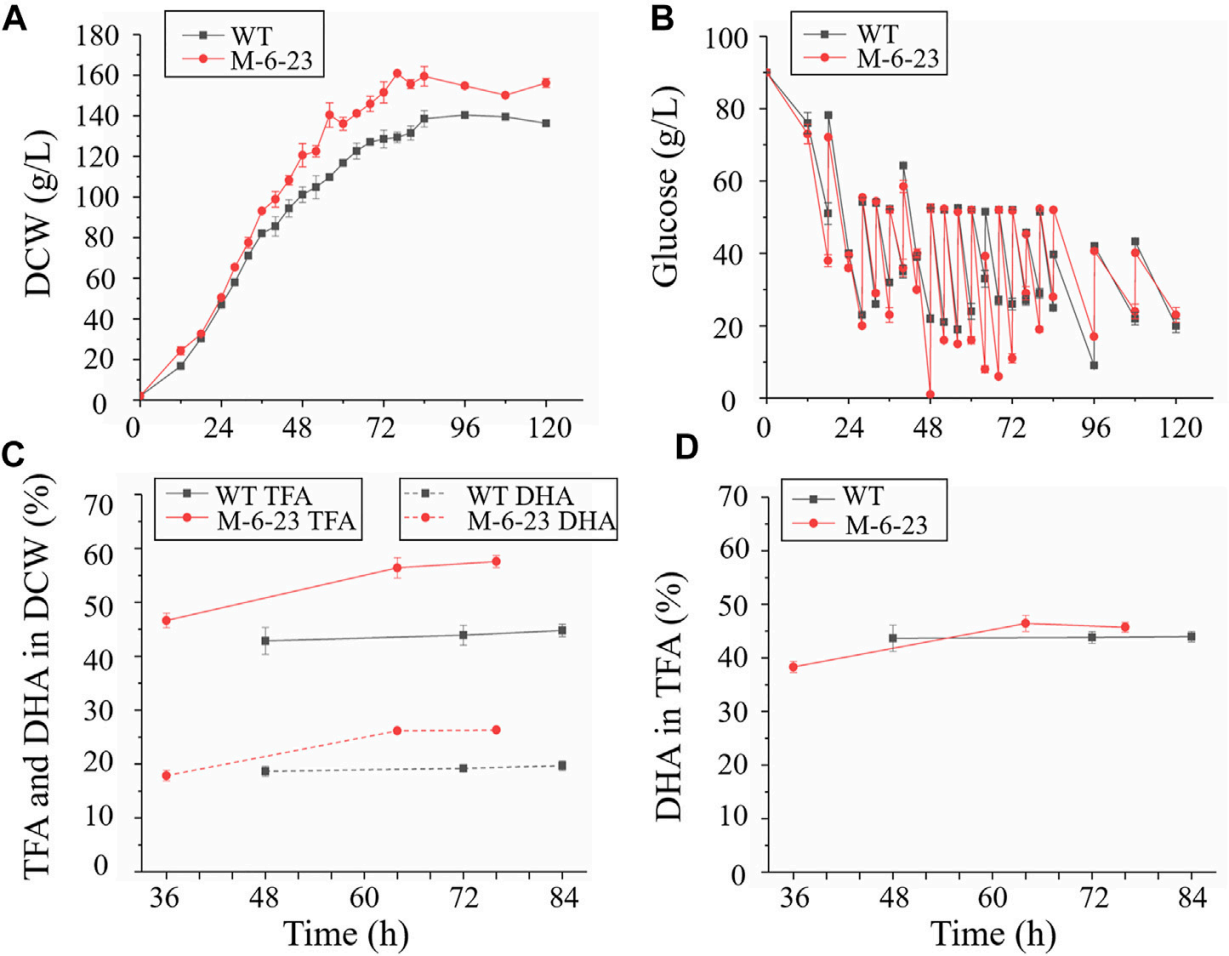
Comparison of biomass accumulation, glucose concentration total fatty acid and DHA content in 5 L fermentors. Black represents wild type (WT); red represents mutant 6–23 (M-6–23), respectively. **(A)** biomass accumulation of wild type and mutant 6–23; **(B)** biomass accumulation of mutant 6–23; **(C)** total fatty acid (TFA) and DHA content in dry cell weight (DCW); **(D)** DHA content in total fatty acid. Error bar represents standard deviation of three biological replicates.

Pulse-feeding glucose fed-batch fermentation was performed in a 5 L fermentor to assess the growth and fatty acid biosynthesis, as well as application potential of mutant 6–23. The glucose concentration was maintained at 20–50 g/L (Figure 5B). Both mutant 6–23 and the wild type entered exponential growth phase at 12 h, and the specific growth rate of mutant 6–23 was *μ* = 0.028 h^−1^, while it was *μ* = 0.023 h^−1^ for wild type during exponential growth phase. Therefore, mutant 6–23 grew faster than the wild type, and mutant 6–23 approached the stationary phase at 76 h, earlier than the 84 h for the wild type (Figure 5A). The final biomass accumulation of mutant 6–23 was 154.8 ± 1.2 g/L at 96 h, 10% higher than that of the wild type (140.4 ± 0.9 g/L).

As mutant 6–23 achieved the maximal growth approximately 8 h earlier than that of the wild type, cells of mutant 6–23 were harvested at 36, 64, and 76 h, while the cells of the wild type were collected at 48, 72, and 84 h, which were corresponding to middle and late exponential and stationary phases of each of their growth phases, respectively. They were then subjected to total fatty acid and DHA analysis. As shown in Figure 5C, the total fatty acid and DHA content of mutant 6–23 were significantly elevated by 28.6 and 33.7% at stationary phase, respectively, while DHA ratio in total fatty acid was not enhanced in mutant 6–23 (Figure 5D). The results showed that DHA concentration and productivity of mutant 6–23 were 42.4 g/L and 557.5 mg/L/h at stationary phase, respectively. The significant enhancement of biomass and DHA content in DCW collectively increased DHA concentration and productivity by 55.3 and 71.6% in mutant 6–23. DHA production by different *Schizochytrium* or *Aurantiochytrium* strains reported so far were summarized in Table 1, which showed that mutant 6–23 achieved the highest synthesis based on DHA concentration and productivity among all studies reported so far.

**TABLE 1:** Comparison of DHA production by *Schizochytrium* strains in different reports.

*Schizochytrium* sp.	Experimental conditions	Yield (g DHA per g DCW)	Fermentation time (h)	Dry cell weight (g/L)	DHA titer (g/L)	DHA productivity (mg/L/h)	References
CCTCC M209059	Fed-batch culture using glucose in 10-L fermentor	0.253	120	67.21	17.02	146.7	Qu et al. (2013b)
HX-308-ALE Endpoint strain	Fed-batch culture using glucose in 5 L fermentor	0.313	120	84.34	26.40	220.04	Sun et al. (2016)
HX-308	Fed-batch culture using glucose and cane molasses in 50 L fermentor	0.194	120	78.26	15.22	126.83	Yin et al. (2019)
ATCC 20888 AB-610	Fed-batch culture using glucose in 7.5 L fermentor	0.191	120	59.96	11.44	95.33	Zhao et al. (2017)
LU301	Culture in 1,000 ml baffled flasks with glucose	0.293	120	84.34	24.74	241.5	Ling et al. (2015)
PQ6	Fed-batch culture using glucose in 30 L fermentor	0.056	96	105.25	5.919	61.66	Hoang et al. (2018)
ATCC 20888	Fed-batch culture using glycerol in 7.5 L fermentor	0.192	96	151.4	28.93	301	Chang et al. (2013)
M-6-23	Fed-batch culture using in 5 L fermentor	0.267	96	154.8	41.35	430.73	This study

## Discussion

ARTP has successfully applied to various marine microorganisms to obtain mutants with better phenotype. For example, enhancing the production capacity of lipid in the oleaginous microalgae *Chlorella pyrenoidosa* (Cao et al., 2017). In addition, improving productivity and the yield of DHA were increased by 1.7- and 1.3-fold in *Crypthecodinium cohnii* (Lv et al., 2020). In this study, *Schizochytrium* ATCC 20888 was treated by ARTP mutagenesis with a new screening approach using EMP and PPP inhibitor, iodoacetic acid and DHEA, as screening agents. This led to identification of three mutants named 6–8, 6–16, and 6–23 with increased biomass and DHA accumulation. LC-MS metabolomic analysis was applied to explore possible mechanisms relevant to enhanced growth and DHA accumulation. The analysis suggested that the upregulated metabolism in PPP and EMP as well as the downregulated TCA cycle might be relevant to the increased growth and DHA biosynthesis in these mutants. At last, mutant 6–23 fermented in 5 L fermentors produced the highest DHA titer and productivity reported in literature.

Metabolic inhibitors have been well studied for screening mutants with improved characterization (Liu et al., 2015). It was reported that EMP, TCA, fatty acid synthase, and ROS are potential metabolic pathways responsible for biomass and DHA production increase in marine microorganisms, and the corresponding targeted inhibitors such as iodoacetic acid, malonic acid, quizalofop-p-ethyl, and 2′, 2′-bipyridine were well developed for screening mutants with increased biomass and lipid production (Lian et al., 2010; Xu et al., 2012; Yuan et al., 2015). However, no report focused on inhibitors targeted for PPP, which has demonstrated to increase lipid accumulation by elevating NADPH supply in marine microorganisms (Cui et al., 2016). In this study, it was the first time to prove dehydroepiandrosterone, an effective inhibitor of PPP, can be used as a screening agent for obtaining mutants with increasing DHA production in *Schizochytrium*. Since enhancing PPP could impair growth in *Aurantiochytrium* sp. SD116 and *Chlorella pyrenoidosa* (Cui et al., 2016; Xue et al., 2018), iodoacetic acid, an inhibitor of EMP pathways, was also added into plates and larger colonies were picked up. These efforts led to discover mutants 6–8, 6–16 and 6–23 with better performance in DHA production.

Samples harvesting for the analysis of cellular metabolites is a challenging task since it may affect the final conclusions. Centrifugation is a widely used technique for harvesting microbes, however, centrifugal forces could lead to the uncontrolled reaction of metabolism during centrifugation (Veyel et al., 2014). There are different views on centrifugation temperature for harvesting cells. Some people argue that cold stress led to change TCA intermediates and organic acids, even its related enzymes in the microalgae (Valledor et al., 2013; Willette et al., 2018), while other think NAD^+^, NADH, NADP^+^, and NADPH show considerable instability at elevated temperature (Gil et al., 2015). Despite the different views mentioned, we argue from the early studies that it will be good to maintain cells at the similar temperature as its cultivation for metabolite, as long as the cells not broken (Li et al., 2015; Diao et al., 2019).

LC/MS revealed that PPP and EMP were both increased in mutants 6–8, 6–16 and 6–23, consistent with increased the activity of phosphoglyceraldehyde dehydrogenase and glucose-6-phosphate dehydrogenase. Although it still needs more evidence that phosphoglyceraldehyde dehydrogenase and glucose-6-phosphate dehydrogenase were likely to play vital roles in biomass and DHA accumulations in *Schizochytrium sp*. Genes encoding phosphoglyceraldehyde dehydrogenase and glucose-6-phosphate dehydrogenase of the wild type and mutant 6–8, 6–16, and 6–23 were PCR amplified and resequencing. However, no mutation site was found inside coding gene, suggesting that other changes, such as mutation of regulatory protein, possibly provoked the up-regulated activity of phosphoglyceraldehyde dehydrogenase and glucose-6-phosphate dehydrogenase, which may worth further investigation in the future. Similar phenomenon was observed in screening *C*. *cohnii* mutants using ARTP mutagenesis combined with acetyl-CoA carboxylase inhibitor sethoxydim (Liu et al., 2017).

As shown in Figure 2, the fatty acid was not enhanced after 48 h cultivation, and while growth was continued until 72 h. Phosphoglyceraldehyde dehydrogenase is involved in EMP pathway, which is provided acetyl-CoA and ATP for fatty acid and biomass accumulation, while glucose-6-phosphate dehydrogenase is a part of PPP pathway, which is for NADPH supply. It was inferred that NADPH needed for fatty acid biosynthesis was decreased since fatty acids content was no longer increased. This led to the decreased the activity of glucose-6-phosphate dehydrogenase. Acetyl-CoA and ATP were continuously needed as biomass was gradually accumulated until 72 h, resulting in increasing the activity of phosphoglyceraldehyde dehydrogenase. The decrease of glucose-6-phosphate dehydrogenase activity is also observed in *Aurantiochytrium sp*. SD116, a closed relationship of *Schizochytrium* (Cui et al., 2016).

Although many similar changes among these strains were observed, there still exist many different metabolic changes among these mutants, notably: 1) The down-regulated ADP-GCS and UDP-GCS suggested that the carbon source flowed from starch or extracellular polysaccharides into lipid or biomass accumulation in mutant 6–23 (Lv et al., 2020); however, UDP-GCS in mutant 6–16 was up-regulated, suggesting different carbon source partition mechanisms from mutant 6–23; 2) It was observed that NADH at 24 and 48 h was up-regulated in mutant 6–23, but not at 48 h in mutant 6–8 and 6–16. The NADH generated by EMP beyond cellular oxidative capacity could lead to overflow metabolism and repress respiratory genes (Vemuri et al., 2007; Bhat et al., 2016). The down-regulated TCA cycles in mutant 6–23 seemed to be consistent with the phenomenon. The possible reasons of down-regulated TCA cycles in mutant 6–8 and 6–16 might obviously different from that of mutant 6–23; 3) It was discovered that acetyl-CoA, the important precursor of lipogenesis, was obviously decreased in mutant 6–23, but not in mutant 6–8 and 6–16. PUFA ratio (DPA + DHA) in total fatty acid profiles were significantly enhanced in mutant 6–23, but not in mutants 6–8 and 6–16. These results suggested the diversity and complexity of the regulation related to growth and DHA accumulation in *Schizochytrium*.

The results from the comparison of different metabolic changes among these mutants could lead to several possible strategies for further increasing DHA production in mutant 6–23: 1) Overexpressing NADH kinase or replacing NAD^+^- glyceraldhyde-3-phosphate dehydrogenase by NADP^+^- glyceraldhyde-3-phosphate dehydrogenase to convert NADH into NADPH in mutant 6–23. Overexpressing the mitochondrial NADH kinase increased the *μ*_*max*_ by 11% in *S*. *cerevisiae* (Hou et al., 2009). Introducing NADP^+^-glyceraldhyde-3-phosphate dehydrogenase to replace NAD^+^-glyceraldhyde-3-phosphate dehydrogenase could improve 17.8–20.0% lipid yields in *Y. lipolytica* (Qiao et al., 2017); 2) Enhancement of acetyl-CoA production by installing non-oxidative glycolytic pathway (NOG). NOG pathway can produce 3 mol acetyl-CoA from 1 mol glucose, which is efficient than EMP. Introducing NOG results in 16.4% higher lipid content and 41% higher dry cell weight in *Y*. *lipolytica* (Qiao et al., 2017). The genetic manipulation platforms, such as electrotransformation, *Agrobacterium tumefaciens* mediated transformation and *Cre*-*loxp* method, have been established in *Schizochytrium* (Cheng et al., 2012; Ren et al., 2015; Sun et al., 2015; Wang et al., 2019). Fully sequencing of mutant 6–23 will provide much more useful information for further investigation, which will be carried out in the near further. Above, these researches make application of above-mentioned strategies feasible in the future.

DHA titer and productivity are important to evaluate the commercial application potential of marine microorganism strains. Before our study, the highest DHA concentration achieved 28.93 g/L, and the highest DHA productivity 301 mg/L/h using *Schizochytrium* ATCC 20888 using glycerol as carbon source (Chang et al., 2013). In this study, DHA titer and productivity were improved to 41.4 g/L and 430.7 mg/L/h respectively by a fermentation of mutant 6–23 with glucose as carbon source. In addition, the maximum yield of glycerol into fatty acid is around 0.10 ± 0.02 g/g, while it is approximately 0.365 g/g of glucose (Chang et al., 2013; Qiao et al., 2017). In all, mutant 6–23 seems a good candidate marine microorganism for industrial production of DHA in the near future.

## Conclusion

A new mutagenesis strategy based on ARTP and two biochemical inhibitors, iodoacetic acid and dehydroepiandrosterone, was applied to *Schizochytrium* ATCC20888. After two rounds of screening, mutants 6–8, 6–16 and 6–23 were obtained. Comparing the starting strain, the growth and total fatty acid content of these strains were significantly elevated, resulting in the DHA concentration increases of 51.9, 45.0, 58.5% in mutants 6–8, 6–16 and 6–23 in shaking flask cultivations, respectively. A targeted LC-MS metabolomic analysis showed that the strengthening metabolism in PPP and EMP as well as attenuating TCA cycles might be related to the increased growth and lipid biosynthesis in *Schizochytrium*. At last, mutant 6–23 was evaluated in a 5-L fermentor, and the results showed that the DHA concentration and productivity of mutant 6–23 were 41.4 g/L and 430.7 mg/L/h in fermentation for 96 h, respectively, which represent the highest reported in literature so far.

## Data Availability

The original contributions presented in the study are included in the article/Supplementary Material, further inquiries can be directed to the corresponding authors.
