# Supplementary material for: Metabolic Analysis of Schizochytrium Mutants With High DHA Content Achieved With ARTP Mutagenesis Combined With Iodoacetic Acid and Dehydroepiandrosterone Screening
Source: Front Bioeng Biotechnol. 2021 Nov 18;9:738052. doi: 10.3389/fbioe.2021.738052 (PMC8637758; doi:10.3389/fbioe.2021.738052)
Supplement: Supplementary file 1 [file DataSheet1.PDF]

Metabolic analysis of *Schizochytrium* mutants with high DHA content achieved with ARTP mutagenesis combined with iodoacetic acid and dehydroepiandrosterone screening

Lei Zeng [1, 3, 4], Yanqi Bi [1, 3, 4], Pengfei Guo [1, 3, 4], Yali Bi [1, 3, 4], Tiantian Wang[1, 3, 4], Liang Dong[1, 3, 4], Fangzhong Wang [1, 2, *], Lei Chen [1, 3, 4], Weiwen Zhang[1, 2, 3, 4, *]

[1] Laboratory of Synthetic Microbiology, School of Chemical Engineering & Technology, Tianjin University, Tianjin, P.R. China; [2] Center for Biosafety Research and Strategy, Tianjin University, Tianjin, P.R. China; [3] Frontier Science Center for Synthetic Biology and Key Laboratory of Systems Bioengineering (MOE), School of Chemical Engineering and Technology, Tianjin University, Tianjin, 300350, People's Republic of China; [4] SynBio Research Platform, Collaborative Innovation Center of Chemical Science and Engineering (Tianjin), P.R. China

* To whom correspondence should be addressed:

Dr. Fangzhong Wang and Prof. Dr. Weiwen Zhang

Laboratory of Synthetic Microbiology

School of Chemical Engineering & Technology

Tianjin University, Tianjin 300072, P. R. China

Tel: 0086-22-2740-6394 ;

Email: fangzhong.wang@tju.edu.cn; and wwzhang8@tju.edu.cn

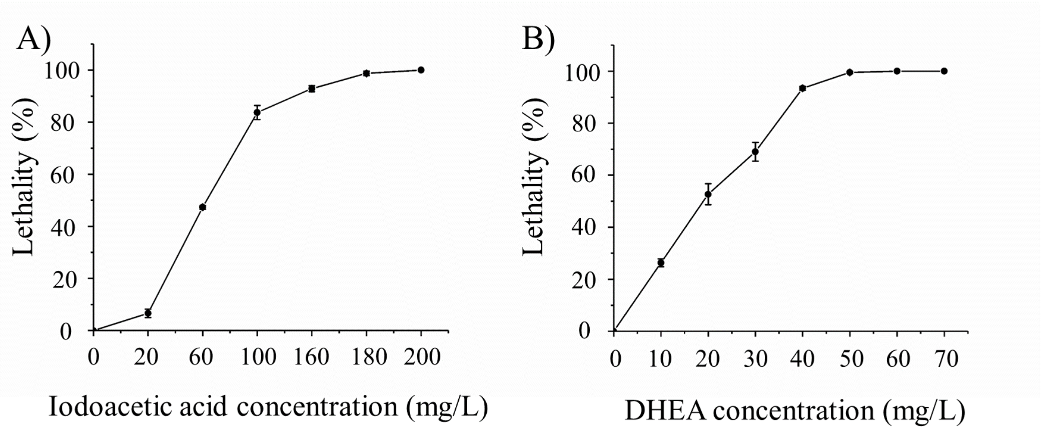

Fig. S1: Sensitivity of *Schizochytrium* ATCC20888 to selected agents. A) iodoacetic acid; **B**) dehydroepiandrosterone (DHEA). Error bar represents standard deviation of three biological replicates.

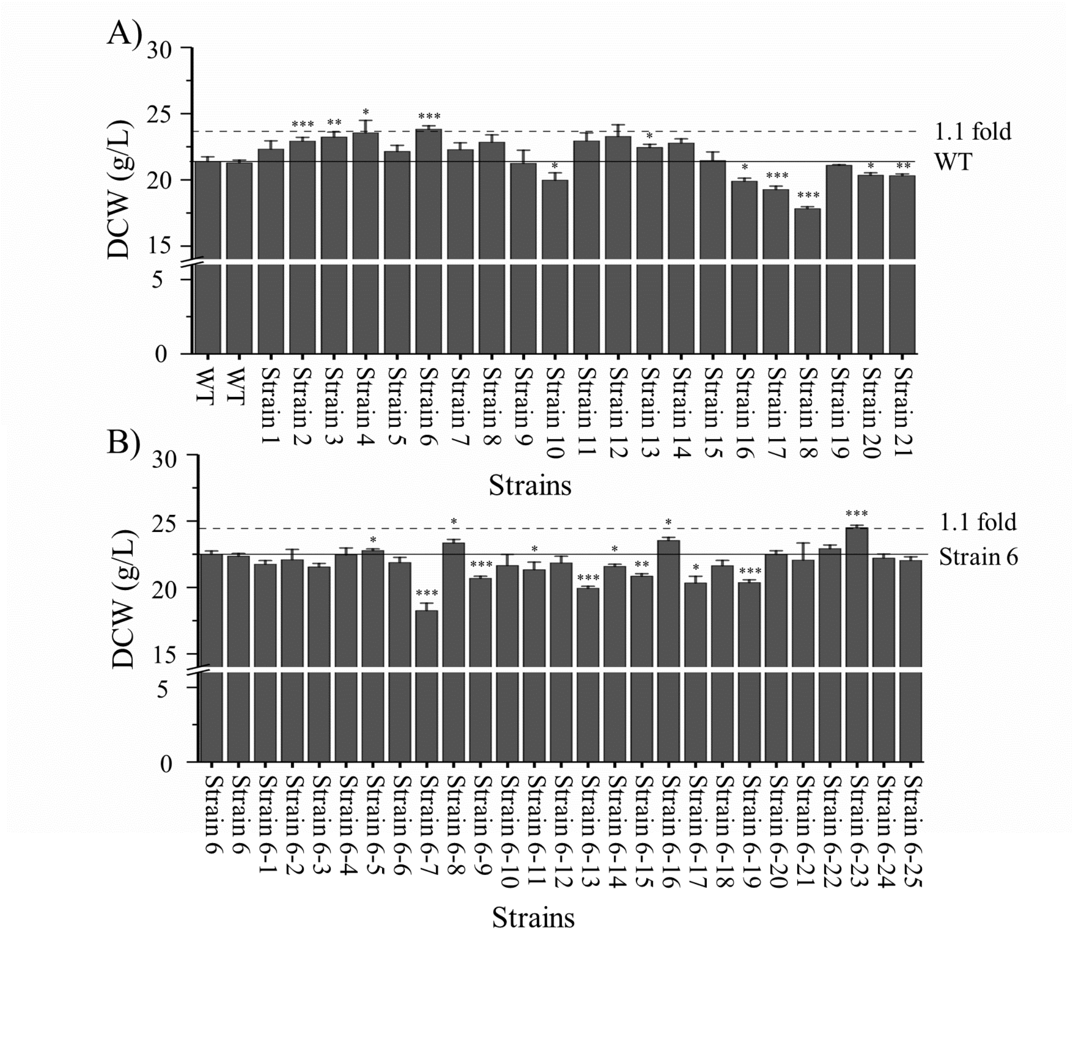

Fig. S2: Comparison of biomass accumulations of different mutants. Error bar represents standard deviation of three biological replicates. Asterisks indicate significant difference between the control and stress treatments based on Student's *t*-tests (∗p < 0.05; ∗∗p < 0.01; ∗∗∗p < 0.005).

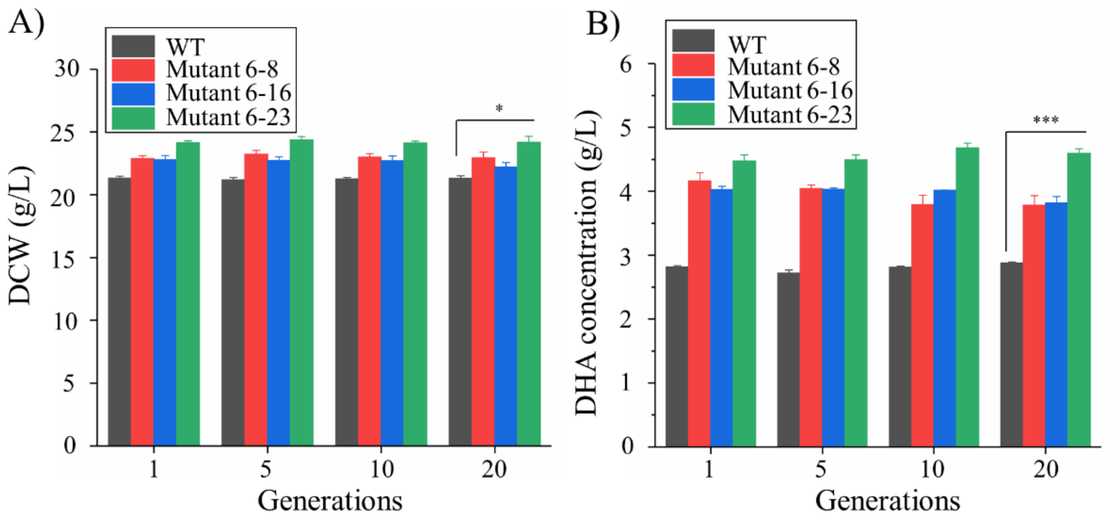

Fig. S3: Comparison of stability of mutants by continous passage. A) Dry cell weight ; B) DHA titer. Black represents the wild type; red represents strain 6-8; blue represents mutant 6-16; green represents mutant 6-23, respectively. Error bar represents standard deviation of three replicates. Asterisks indicate significant difference between the control and stress treatments based on Student's *t*-tests (∗p < 0.05; ∗∗p < 0.01; ∗∗∗p < 0.005).

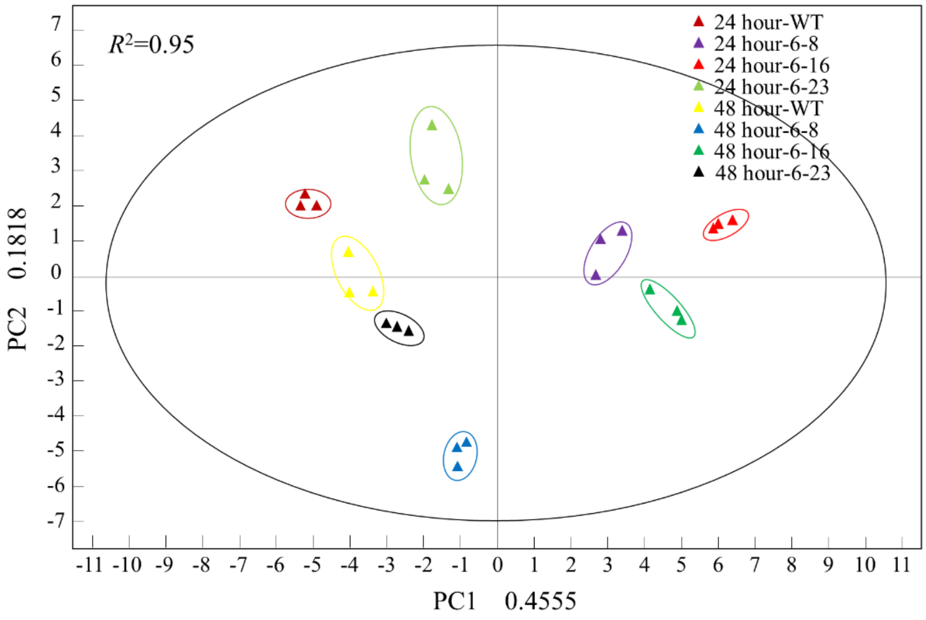

Supplementary Fig. S4. PCA of LC-MS targeted metabolomics. PCA analysis was performed with SIMCA-P11.59 software. Three biological replicates were carried out.

Table S1. Several screening agents used in *Schizochytrium*

Schizochytrium sp.	Screening agent	Target	References
HX-308M	Iodoacetate acid<br>Malonic acid	Enhancing acetyl-CoA supply	Lian et al., 2010
OUC002<br>OUC007	Sethoxydim	Enhancing malonyl-CoA supply	Xu et al., 2012
D32	2', 2'-bipyridine	Enhancing antioxidant capacity	Yuan et al., 2015

Table S2. Comparison of DPA and DHA titers of wild type and first-round mutants. The data are the mean ± standard deviation (n = 3).

**Strains**	**DPA titer (g/L)**	**DHA titer (g/L)**
WT	0.99±0.02	2.89±0.02
1	0.93±0.25	2.95±0.28
2	0.76±0.13	1.98±0.33
3	0.63±0.16	2.89±0.19
4	0.79±0.06	1.99±0.12
6	0.76±0.23	3.92±0.12
11	0.86±0.14	2.23±0.19
12	0.68±0.10	3.41±0.14
13	0.69±0.04	1.88±0.02
14	0.70±0.09	3.29±0.08

Table S3 Comparison of DPA and DHA titers of wild type and second-round mutants. The data are the mean ± standard deviation (n = 3).

**Strains**	**DPA production (g/L)**	**DHA production (g/L)**
WT	0.99±0.02	2.89±0.02
6-2	0.75±0.16	3.27±0.13
6-4	1.13±0.12	3.88±0.06
6-5	0.67±0.07	3.88±0.05
6-8	0.61±0.07	4.39±0.32
6-16	0.59±0.09	4.19±0.22
6-20	1.40±0.19	3.53±0.24
6-22	1.57±0.12	3.42±0.16
6-23	1.36±0.17	4.58±0.13

Table S4. LC-MS metabolomic data set of WT and mutants at different time points. Three biological replicates.

24 hour	wt-1	wt-2	wt-3	6-8-1	6-8-2	6-8-3	6-16-1	6-16-2	6-16-3	6-23-1	6-23-2	6-23-3
NADPH	400	386	414	961	887	888	1472	1638	1771	731	875	718
$NADP^+$	3855	3746	3872	9759	9736	9572	12448	12377	11121	4707	5101	4809
NADH	27	30	24	41	49	40	35	44	46	44	35	43
NAD^+	10453	10626	10184	7708	8009	8015	9085	9156	8156	9807	10873	11863
ADP-GCS	158	148	187	137	113	111	91	97	115	103	115	190
UDP-GCS	23211	22871	22382	54430	58267	55988	51090	54158	49928	15066	16905	17250
ATP	9313	9251	9289	11878	11987	11676	14325	14131	13165	9859	9772	9947
ADP	12242	11590	11682	14707	14616	15927	19995	18235	9910	9673	10620	10146
CoA	852	812	842	1657	1631	1411	1331	1524	1531	1198	1355	1250
AMP	50311	51876	52906	51935	52131	52212	65158	61682	61655	55747	57847	57024
FBP	641	674	646	996	913	920	2105	2157	2152	1281	1402	1365
F6P	13788	14124	14886	24117	23229	23276	30982	31123	28220	17191	17142	18037
R5P	532	544	514	1789	1621	1608	2164	2418	2924	1556	1571	1893
E4P	1877	1971	2037	4090	4840	3816	4530	4596	4307	2987	3217	3154
CIT	183	183	184	145	137	138	125	148	137	170	172	174
3-PG	1250	1150	1160	5648	5829	6552	8369	8387	8314	3085	3288	3104
1,3-PG	723	738	746	3842	3916	3951	4189	3789	4256	2090	2600	2587
GAP	712	738	778	4179	4275	4113	5432	5696	5345	1611	1827	1679
PEP	196	187	196	271	311	296	234	312	260	284	365	372
Glutamic acid	46022	45784	45995	19256	19906	19727	26157	25186	24922	39121	43374	40640
AKG	1292	1303	1282	1259	1224	1198	1807	1887	1902	1509	1822	1549

Table S3 (continued)

24 hour	wt-1	wt-2	wt-3	6-8-1	6-8-2	6-8-3	6-16-1	6-16-2	6-16-3	6-23-1	6-23-2	6-23-3
MAL	13972	14301	13643	11835	10189	10458	11235	10892	10996	14886	11362	16053
SUC	21645	21251	22039	19909	20232	19190	21440	19708	21027	21027	19086	19559
FUM	5117	5084	5150	3409	3359	3318	2805	2983	2856	4662	4707	5215
PYR	865	844	886	1397	1448	1454	1512	1498	1686	1054	1159	1038
Ac-CoA	418	354	375	1524	1532	1559	1369	1273	1625	240	263	236
Phenylanine	11215	11150	11182	18380	16146	22456	21676	21591	22431	16103	14162	15329
Lysine	29081	27407	27834	34196	34643	18113	49256	46629	54801	32022	35325	36290
Aspartic acid	45015	41155	40131	72869	84617	57171	45672	51601	62461	45635	49867	48240
Leucine	8115	7115	6712	7324	10076	8984	11125	9676	11414	4731	4651	4280
Valine	3927	4015	4137	7057	8947	6944	7210	6093	8478	8975	8040	8890
Serine	49199	51911	50923	47414	48665	41754	41834	41731	50058	52571	47544	50920
Alanine	4591	4546	4481	8511	8708	7627	10086	8894	11780	5099	5089	5567

Table S3 (continued)

48 hour	wt-1	wt-2	wt-3	6-8-1	6-8-2	6-8-3	6-16-1	6-16-2	6-16-3	6-23-1	6-23-2	6-23-3
NADPH	483	371	339	646	552	574	1150	864	1003	629	681	647
$NADP^+$	4052	3984	4012	4968	4631	4772	12077	13206	13222	5414	4892	5001
NADH	30	32	31	20	22	29	28	18	17	43	45	44
NAD^+	12308	10236	11212	7963	6050	7027	8835	9375	8821	8855	10067	9411
ADP-GCS	122	102	100	48	54	49	79	89	87	52	81	75
UDP-GCS	51217	55030	52231	20209	20793	19976	52131	55192	54213	33042	32831	31342
ATP	7204	6420	7212	9233	7175	8211	12453	16101	14021	9716	9504	9701
ADP	7352	8398	8211	13411	11211	12423	15813	18853	17212	7129	7321	7213
CoA	1033	884	1121	1476	1446	1591	1453	1385	1548	1333	1289	1314
AMP	40196	36233	38312	29574	30590	31011	62721	50255	56121	35537	30290	32012
FBP	938	894	982	1275	1415	1321	2018	2257	2122	877	754	782
F6P	17076	17898	16812	13999	11134	12312	31763	38488	35123	17289	17533	17921
R5P	712	608	652	1284	986	988	2488	2169	2231	1680	1758	1765
E4P	2947	2475	2761	2159	1938	2013	4854	5012	4813	2152	2398	2236
CIT	216	226	167	138	151	156	125	112	121	221	243	265
3-PG	1223	1016	1121	3446	3216	3321	7468	7142	7321	3061	3051	3021
1,3-PG	872	817	812	2178	1912	1921	3220	4106	3543	2037	1855	1843
GAP	1126	995	954	1332	1268	1187	4719	3901	4329	2144	1991	2065
PEP	92	90	95	95	94	86	229	285	230	94	95	100
Glutamic acid	39154	40539	41211	13070	13878	14328	24296	20032	22924	51131	48618	46725
AKG	1330	1273	1088	1462	1535	1623	1617	1575	1743	1697	1705	1607

Table S3 (continued)

48 hour	wt-1	wt-2	wt-3	6-8-1	6-8-2	6-8-3	6-16-1	6-16-2	6-16-3	6-23-1	6-23-2	6-23-3
MAL	12858	9195	10211	8423	8895	8721	12131	10889	10212	8006	7886	10112
SUC	19410	21847	20515	16227	15910	16121	21074	20681	21290	20474	18882	17308
FUM	3960	3167	4070	3036	3012	2912	2912	2820	2915	3217	3070	3511
PYR	808	864	878	1483	1624	1512	1336	1320	1206	1088	1001	973
Ac-CoA	529	612	620	2844	2802	3003	2606	4628	4312	409	411	354
Phenylalanine	19857	22651	22372	20017	20651	18049	25121	23121	28631	17887	20396	21314
Lysine	29396	25005	26326	17421	15639	18922	27719	22951	24824	44477	44430	48113
Aspartic acid	49392	44144	41497	42011	39306	41705	38247	39447	45248	28402	31245	25131
Leucine	8989	8146	10631	7660	11270	8031	7083	8484	6445	4673	5559	5651
Valine	5831	5525	5342	3493	3413	3140	4089	3991	5047	4913	4917	5124
Serine	40518	37827	40677	57846	51886	56071	45080	52592	43224	55821	54121	55358
Alanine	5024	4712	5120	6047	5899	6441	6180	6896	6793	4740	5353	5723
